# Supplementary figures and images for: Spatial variations of microbial communities in abyssal and hadal sediments across the Challenger Deep
Source: PeerJ. 2019 May 17;7:e6961. doi: 10.7717/peerj.6961 (PMC6526897; doi:10.7717/peerj.6961)

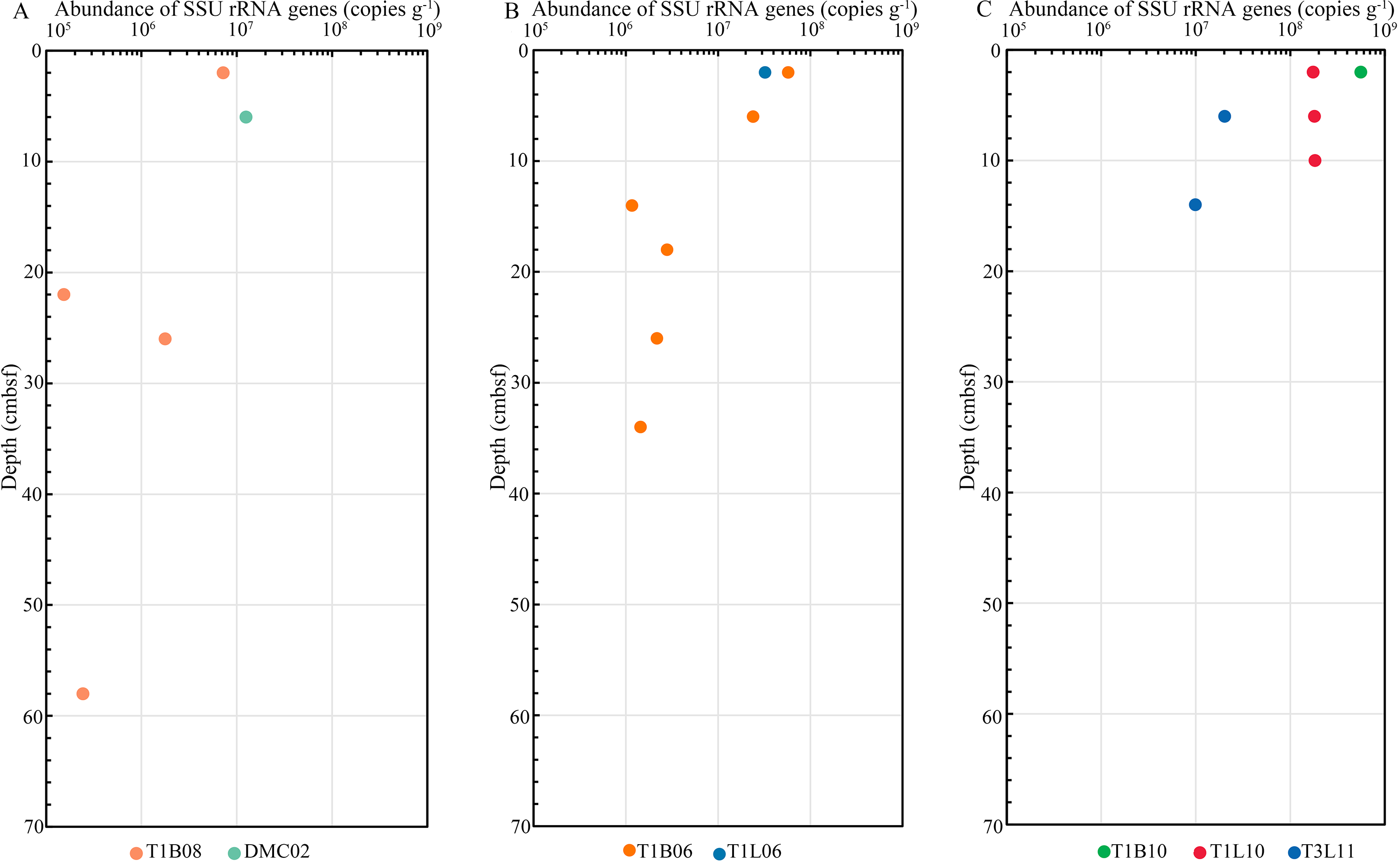

Supplement: Supplemental Information 1 — Copy numbers of whole prokaryotic SSU rRNA genes (copies g−1 sediment) in sediment cores obtained from the Challenger Deep. (A: the northern slope. B: the southern slope. C: the trench-axis). [file peerj-07-6961-s001.png]

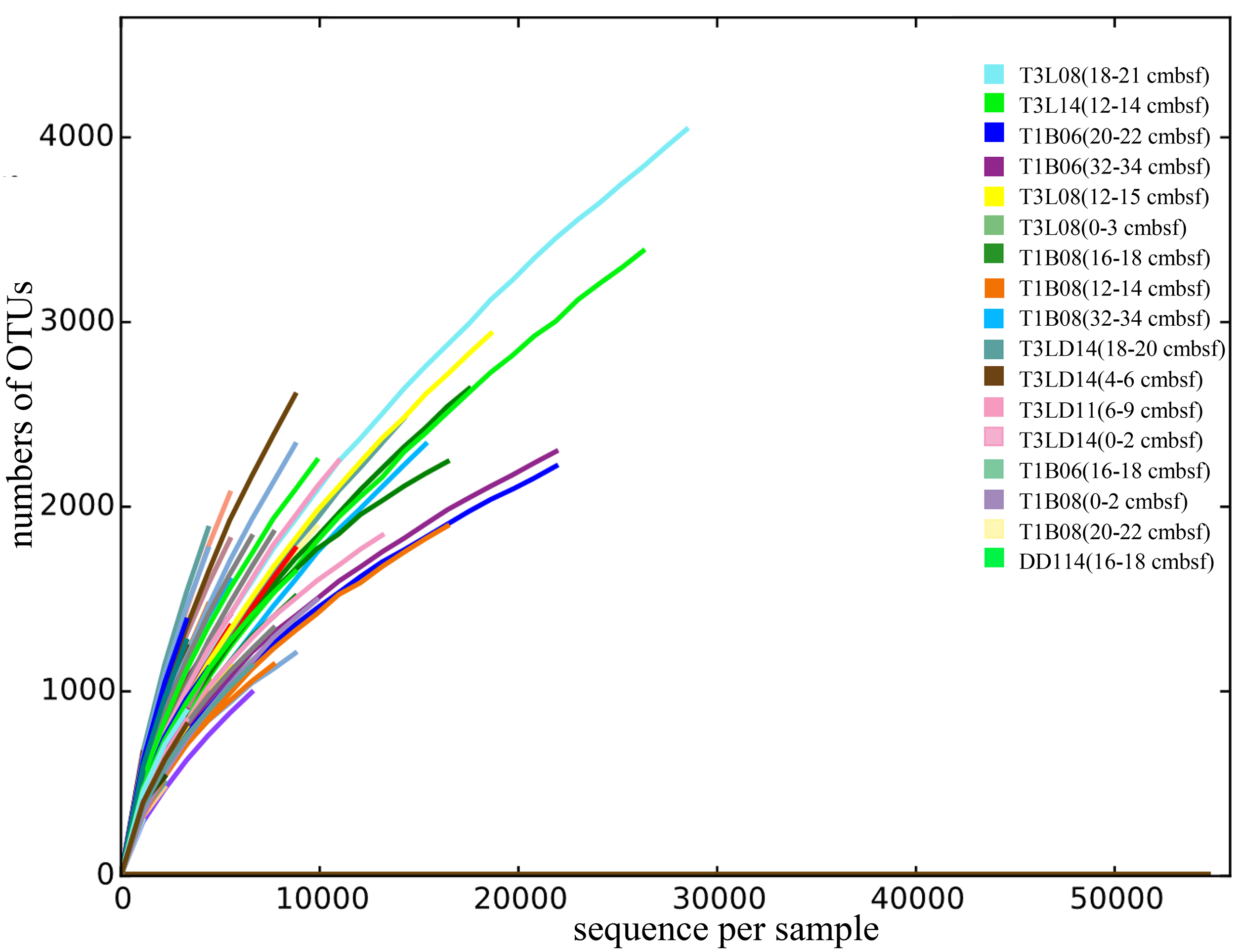

Supplement: Supplemental Information 2 — Curves were calculated with RDP at 3% of dissimilarity. In total 95 sediment samples from Challenger Deep were presented in different curves. Seventeen labels for sample reads number above 10,000 were listed on the top right. [file peerj-07-6961-s002.png]

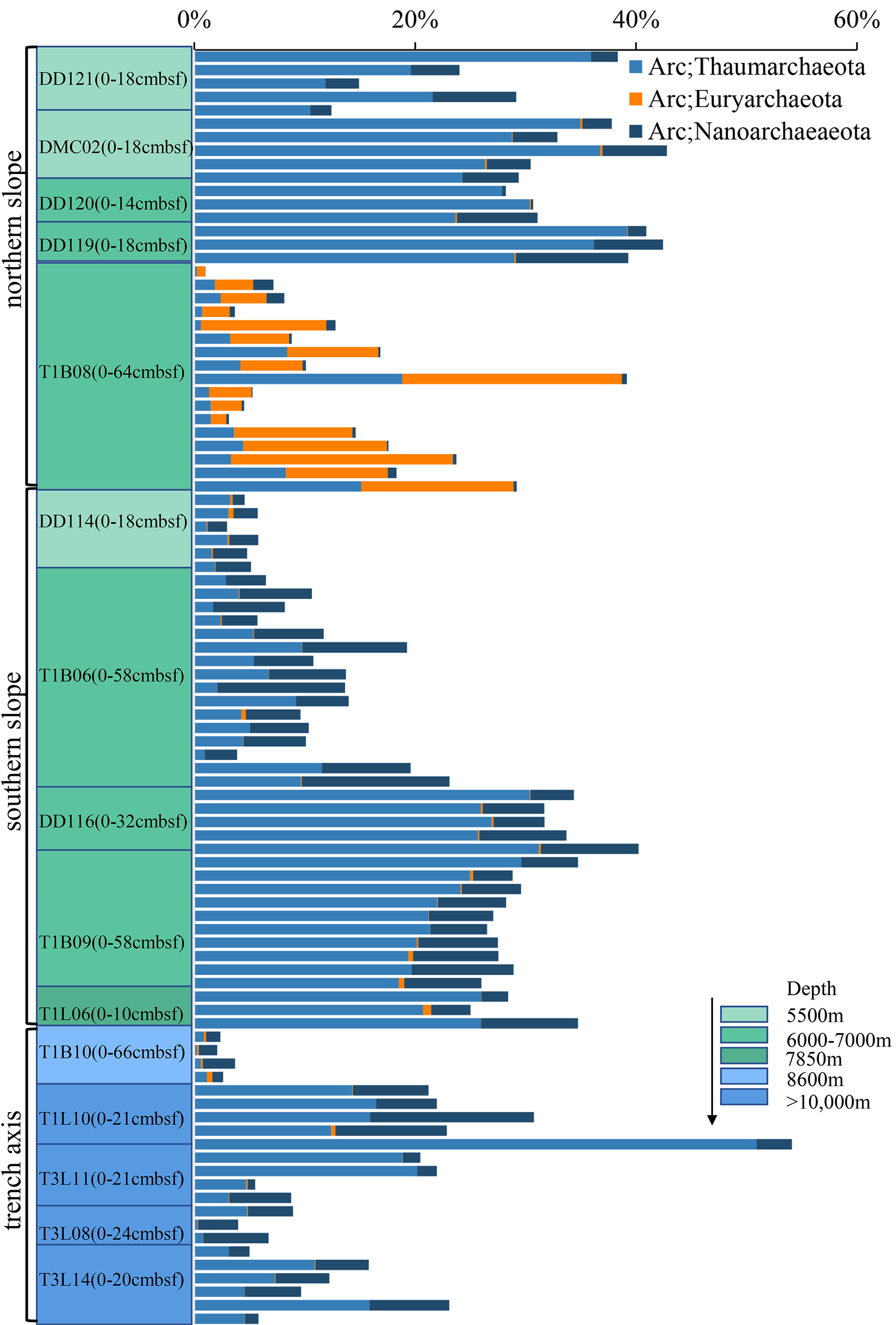

Supplement: Supplemental Information 3 — The microbial communities were revealed based on sequencing of 16S rRNA gene amplicons and classification at the phylum level using the RDP classifier against the SILVA 132 database. The core lengths were indicated in the brackets behind the sample ID. [file peerj-07-6961-s003.png]

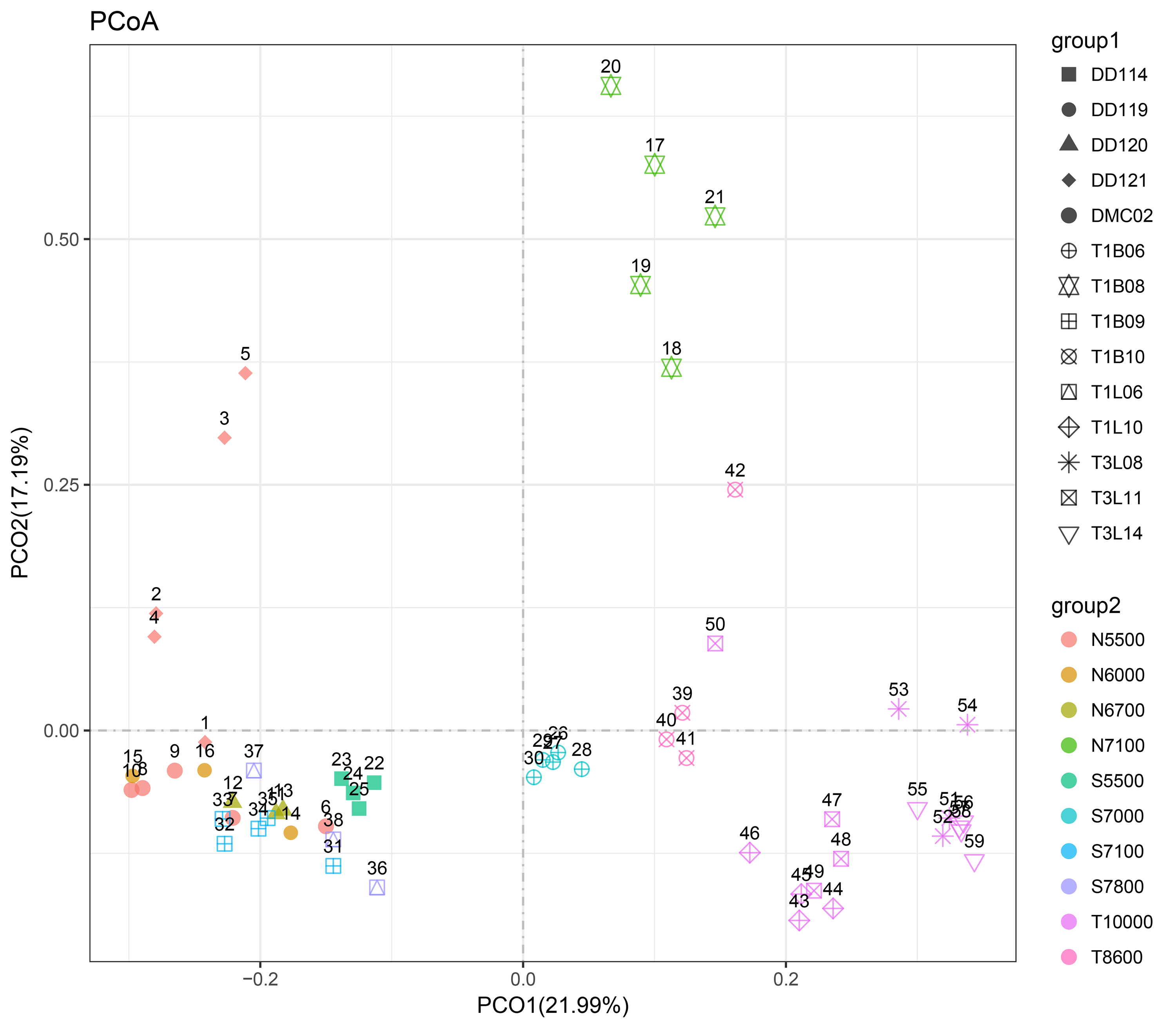

Supplement: Supplemental Information 4 — The percentages of genera in the communities were used for calculation of Bray–Curtis dissimilarities and then a PCoA plot. Color code indicates the different water depths of the samples while different symbols distinguish the samples from each other (simple IDs refer to Table S3). [file peerj-07-6961-s004.png]
