# Supplementary material for: Spatial variations of microbial communities in abyssal and hadal sediments across the Challenger Deep
Source: PeerJ. 2019 May 17;7:e6961. doi: 10.7717/peerj.6961 (PMC6526897; doi:10.7717/peerj.6961)
Supplement: Supplemental Information 5 — # Sediment samples collected by Jiaolong manned submersible. All the sediment samples (water depths > 10,000 m) were obtained by a hadal lander. cmbsf: centimeter below seafloor. [file peerj-07-6961-s005.docx]

**Table S1.** Information of samples collected from the Challenger Deep.

| Cruise | Sample ID | Sampling method | Latitude  [degree North] | Longitude  [degree East] | Depth  [m] | core length[cmbsf] |
| --- | --- | --- | --- | --- | --- | --- |
| DY37II | DMC02 | push core | 11.764 | 141.976 | 5481 | 34 |
| DY37II | DD121# | push core | 11.801 | 142.117 | 5533 | 27 |
| DY37II | DD120# | push core | 11.582 | 141.879 | 6706 | 26 |
| DY37II | DD119# | push core | 11.665 | 142.249 | 6016 | 19 |
| TS01 | T1B08 | box core | 11.602 | 142.228 | 7143 | 64 |
| DY37II | DD114# | push core | 10.851 | 141.950 | 5464 | 28 |
| TS01 | T1B06 | box core | 11.039 | 142.304 | 7022 | 60 |
| TS01 | T1B09 | box core | 10.994 | 141.994 | 7121 | 64 |
| TS01 | T1L06 | push core | 11.091 | 142.073 | 7850 | 15 |
| TS01 | T1B10 | box core | 11.195 | 141.812 | 8638 | 66 |
| TS01 | T1L10 | push core | 11.328 | 142.202 | 10953 | 25 |
| TS03 | T3L11 | push core | 11.325 | 142.191 | 10908 | 22 |
| TS03 | T3L08 | push core | 11.327 | 142.194 | 10909 | 20 |
| TS03 | T3L14 | push core | 11.325 | 142.189 | 10911 | 18 |

# Sediment samples collected by Jiaolong manned submersible. All the sediment samples (water depths > 10,000 m) were obtained by a hadal lander. cmbsf: centimeter below seafloor.
