# Supplementary material for: Spatial variations of microbial communities in abyssal and hadal sediments across the Challenger Deep
Source: PeerJ. 2019 May 17;7:e6961. doi: 10.7717/peerj.6961 (PMC6526897; doi:10.7717/peerj.6961)
Supplement: Supplemental Information 6 — 16S rRNA gene amplicons for 95 sediment layers of 14 sediment samples were sequenced. The observed OTU number, Chao1 and Shannon index were determined at a dissimilarity level of 3% and normalized to the smallest library size (i.e. 1,143 reads). [file peerj-07-6961-s006.docx]

**Table S2.** Statistics of sequencing reads of 16S rRNA gene amplicons.

|  | Sample ID | Qualified reads | Total reads | | | Normalized | | | | |  |
| --- | --- | --- | --- | --- | --- | --- | --- | --- | --- | --- | --- |
|  |  |  | OTUs | chao1 | Shannon | OTUs | | chao1 | | Shannon | |
| 1 | DD121(0-2 cmbsf) | 2281 | 1037 | 2983 | 8.95 | 617 | 2091 | | 8.49 | |  |
| 2 | DD121(4-6 cmbsf) | 1765 | 608 | 2053 | 8.60 | 608 | 2053 | | 8.60 | |  |
| 3 | DD121(8-10 cmbsf) | 2850 | 821 | 2033 | 8.27 | 503 | 1533 | | 7.91 | |  |
| 4 | DD121(12-14 cmbsf) | 1978 | 546 | 1517 | 8.26 | 546 | 1517 | | 8.26 | |  |
| 5 | DD121(16-18 cmbsf) | 1631 | 391 | 1087 | 7.24 | 391 | 1087 | | 7.24 | |  |
| 6 | DMC02(0-2 cmbsf) | 2069 | 667 | 2305 | 8.76 | 667 | 2305 | | 8.76 | |  |
| 7 | DMC02(4-6 cmbsf) | 4193 | 1385 | 3412 | 9.30 | 623 | 2003 | | 8.60 | |  |
| 8 | DMC02(8-10 cmbsf) | 4988 | 1471 | 3707 | 9 | 532 | 1969 | | 8 | |  |
| 9 | DMC02(12-14 cmbsf) | 4232 | 1325 | 3026 | 9.12 | 610 | 1987 | | 8.51 | |  |
| 10 | DMC02(16-18 cmbsf) | 3773 | 1385 | 3355 | 9.34 | 630 | 2088 | | 8.65 | |  |
| 11 | DD120(0-2 cmbsf) | 1187 | 382 | 862 | 7.45 | 382 | 862 | | 7.45 | |  |
| 12 | DD120(4-6 cmbsf) | 4290 | 902 | 1915 | 8.08 | 438 | 1250 | | 7.61 | |  |
| 13 | DD120(12-14 cmbsf) | 1753 | 458 | 1009 | 7.84 | 458 | 1009 | | 7.84 | |  |
| 14 | DD119(0-2 cmbsf) | 2361 | 756 | 1882 | 8.01 | 471 | 1369 | | 7.67 | |  |
| 15 | DD119(12-14 cmbsf) | 1143 | 978 | 7.38 | 410 | 978 | 7.38 | | 410 | |  |
| 16 | DD119(16-18 cmbsf) | 1324 | 415 | 1029 | 7.42 | 415 | 1029 | | 7.42 | |  |
| 17 | T1B08(0-2 cmbsf) | 10117 | 1496 | 2776 | 6.89 | 334 | 1226 | | 6.27 | |  |
| 18 | T1B08(4-6 cmbsf) | 3199 | 538 | 882 | 8.22 | 396 | 656 | | 7.99 | |  |
| 19 | T1B08(8-10 cmbsf) | 5426 | 1126 | 1943 | 8.58 | 483 | 1244 | | 7.97 | |  |
| 20 | T1B08(12-14 cmbsf) | 16900 | 1895 | 3473 | 6.34 | 329 | 1122 | | 5.68 | |  |
| 21 | T1B08(16-18 cmbsf) | 17190 | 2244 | 3112 | 7.70 | 409 | 1419 | | 6.90 | |  |
| 22 | T1B08(20-22 cmbsf) | 10044 | 1892 | 4159 | 8.04 | 420 | 1388 | | 7.31 | |  |
| 23 | T1B08(24-26 cmbsf) | 4904 | 1250 | 4152 | 7.83 | 438 | 1873 | | 7.26 | |  |
| 24 | T1B08(28-30 cmbsf) | 7893 | 1605 | 4546 | 7.65 | 407 | 1484 | | 7.01 | |  |
| 25 | T1B08(32-34 cmbsf) | 16302 | 2339 | 5609 | 7.34 | 344 | 1331 | | 6.49 | |  |
| 26 | T1B08(36-38 cmbsf) | 4699 | 1062 | 2828 | 7.98 | 401 | 1254 | | 7.43 | |  |
| 27 | T1B08(40-42 cmbsf) | 4465 | 1067 | 2698 | 7.86 | 413 | 1282 | | 7.32 | |  |
| 28 | T1B08(44-46 cmbsf) | 6764 | 995 | 2481 | 6.85 | 295 | 972 | | 6.40 | |  |
| 29 | T1B08(48-50 cmbsf) | 5328 | 1118 | 3376 | 7.44 | 405 | 1690 | | 6.92 | |  |
| 30 | T1B08(52-54 cmbsf) | 5084 | 1101 | 3377 | 7.36 | 400 | 1472 | | 6.84 | |  |
| 31 | T1B08(56-58 cmbsf) | 4011 | 890 | 2971 | 7.36 | 394 | 1460 | | 6.89 | |  |
| 32 | T1B08(60-62 cmbsf) | 5236 | 985 | 2537 | 7.66 | 373 | 1277 | | 7.14 | |  |
| 33 | T1B08(62-64 cmbsf) | 8825 | 1774 | 4902 | 7.39 | 374 | 1529 | | 6.61 | |  |
| 34 | DD114(0-2 cmbsf) | 4459 | 1883 | 4631 | 9.85 | 674 | 2539 | | 8.91 | |  |
| 35 | DD114(4-6 cmbsf) | 5831 | 2074 | 4969 | 9.67 | 629 | 2380 | | 8.66 | |  |
| 36 | DD114(6-8 cmbsf) | 9403 | 2337 | 5750 | 9.37 | 546 | 1739 | | 8.39 | |  |
| 37 | DD114(10-12 cmbsf) | 9438 | 2606 | 5568 | 9.67 | 600 | 2196 | | 8.57 | |  |
| 38 | DD114(12-14 cmbsf) | 7139 | 1839 | 3962 | 9.23 | 541 | 1632 | | 8.35 | |  |
| 39 | DD114(16-18 cmbsf) | 10036 | 2251 | 4845 | 9.25 | 527 | 1632 | | 8.33 | |  |
| 40 | T1B06(0-2 cmbsf) | 5518 | 1353 | 3528 | 8.35 | 447 | 1500 | | 7.70 | |  |
| 41 | T1B06(4-6 cmbsf) | 1486 | 442 | 1253 | 7.80 | 442 | 1253 | | 7.80 | |  |
| 42 | T1B06(8-10 cmbsf) | 9423 | 1207 | 2563 | 8.02 | 376 | 895 | | 7.45 | |  |
| 43 | T1B06(12-14 cmbsf) | 3286 | 479 | 1001 | 7.17 | 323 | 678 | | 6.96 | |  |
| 44 | T1B06(16-18 cmbsf) | 10552 | 1447 | 3229 | 8.37 | 422 | 988 | | 7.73 | |  |
| 45 | T1B06(20-22 cmbsf) | 22696 | 2220 | 4597 | 8.07 | 391 | 999 | | 7.31 | |  |
| 46 | T1B06(24-26 cmbsf) | 8532 | 1146 | 2512 | 7.94 | 374 | 869 | | 7.38 | |  |
| 47 | T1B06(28-30 cmbsf) | 9233 | 1515 | 2924 | 8.14 | 423 | 1139 | | 7.48 | |  |
| 48 | T1B06(32-34 cmbsf) | 21991 | 2300 | 4487 | 8.33 | 410 | 1029 | | 7.57 | |  |
| 49 | T1B06(36-38 cmbsf) | 5694 | 1142 | 2517 | 7.12 | 385 | 1202 | | 6.53 | |  |
| 50 | T1B06(38-40 cmbsf) | 3483 | 893 | 1693 | 8.37 | 458 | 1111 | | 7.94 | |  |
| 51 | T1B06(42-44 cmbsf) | 3634 | 844 | 1910 | 8.21 | 431 | 1040 | | 7.80 | |  |
| 52 | T1B06(44-46 cmbsf) | 6920 | 1140 | 2643 | 7.83 | 374 | 917 | | 7.28 | |  |
| 53 | T1B06(48-50 cmbsf) | 4233 | 820 | 1959 | 7.92 | 398 | 1079 | | 7.50 | |  |
| 54 | T1B06(54-56 cmbsf) | 8085 | 1344 | 2979 | 8.08 | 410 | 1090 | | 7.44 | |  |
| 55 | T1B06(56-58 cmbsf) | 9570 | 1648 | 3221 | 8.62 | 474 | 1201 | | 7.89 | |  |
| 56 | T1B09(0-2 cmbsf) | 5106 | 1772 | 4529 | 9.53 | 644 | 2368 | | 8.65 | |  |
| 57 | T1B09(4-6 cmbsf) | 5881 | 1823 | 4094 | 9.31 | 601 | 2010 | | 8.46 | |  |
| 58 | T1B09(8-10 cmbsf) | 1742 | 561 | 1673 | 8.19 | 561 | 1673 | | 8.19 | |  |
| 59 | T1B09(12-14 cmbsf) | 2073 | 609 | 1965 | 8.41 | 609 | 1965 | | 8.41 | |  |
| 60 | T1B09(16-18 cmbsf) | 1771 | 568 | 1623 | 8.14 | 568 | 1623 | | 8.14 | |  |
| 61 | T1B09(20-22 cmbsf) | 1648 | 547 | 1416 | 8.11 | 547 | 1416 | | 8.11 | |  |
| 62 | T1B09(24-26 cmbsf) | 2906 | 940 | 2474 | 8.73 | 576 | 1719 | | 8.30 | |  |
| 63 | T1B09(28-30 cmbsf) | 2761 | 951 | 2488 | 8.74 | 582 | 1795 | | 8.33 | |  |
| 64 | T1B09(32-34 cmbsf) | 2711 | 934 | 2177 | 8.89 | 585 | 1612 | | 8.49 | |  |
| 65 | T1B09(36-38 cmbsf) | 3885 | 1102 | 2405 | 8.78 | 542 | 1418 | | 8.25 | |  |
| 66 | T1B09(40-42 cmbsf) | 1593 | 579 | 1626 | 8.38 | 579 | 1626 | | 8.38 | |  |
| 67 | T1B09(44-46 cmbsf) | 5654 | 1605 | 3101 | 9.11 | 560 | 1617 | | 8.32 | |  |
| 68 | T1B09(48-50 cmbsf) | 4054 | 1163 | 2282 | 9.07 | 571 | 1418 | | 8.45 | |  |
| 69 | T1B09(52-54 cmbsf) | 2916 | 801 | 1694 | 8.45 | 517 | 1243 | | 8.10 | |  |
| 70 | T1B09(56-58 cmbsf) | 4298 | 1084 | 2055 | 8.84 | 550 | 1312 | | 8.30 | |  |
| 71 | T1L06.T2(0-2 cmbsf) | 4546 | 1233 | 2447 | 8.86 | 510 | 1277 | | 8.20 | |  |
| 72 | T1L06.T2(4-6 cmbsf) | 4388 | 1114 | 2389 | 8.63 | 482 | 1195 | | 8.05 | |  |
| 73 | T1L06.T2(8-10 cmbsf) | 3351 | 1157 | 2193 | 8.94 | 576 | 1532 | | 8.37 | |  |
| 74 | T1B10(0-2 cmbsf) | 2147 | 433 | 1229 | 7.25 | 433 | 1229 | | 7.25 | |  |
| 75 | T1B10(28-30 cmbsf) | 4097 | 807 | 1567 | 7.91 | 417 | 1009 | | 7.49 | |  |
| 76 | T1B10(44-46 cmbsf) | 1817 | 545 | 1340 | 8.48 | 545 | 1340 | | 8.48 | |  |
| 77 | T1B10(64-66 cmbsf) | 3108 | 784 | 2186 | 7.76 | 475 | 1375 | | 7.39 | |  |
| 78 | T1L10(0-3 cmbsf) | 4338 | 1112 | 2259 | 8.88 | 555 | 1357 | | 8.33 | |  |
| 79 | T1L10(6-9 cmbsf) | 4175 | 1088 | 2416 | 8.65 | 524 | 1515 | | 8.10 | |  |
| 80 | T1L10(12-15 cmbsf) | 6131 | 1413 | 2662 | 8.96 | 538 | 1332 | | 8.24 | |  |
| 81 | T1L10(18-21 cmbsf) | 3854 | 1023 | 2110 | 8.70 | 520 | 1216 | | 8.20 | |  |
| 82 | T3L11(0-3 cmbsf) | 1915 | 303 | 793 | 5.68 | 303 | 793 | | 5.68 | |  |
| 83 | T3L11(6-9 cmbsf) | 13720 | 1846 | 2940 | 8.33 | 427 | 1042 | | 7.58 | |  |
| 84 | T3L11(12-15 cmbsf) | 3203 | 507 | 971 | 7.45 | 345 | 682 | | 7.22 | |  |
| 85 | T3L11(18-21 cmbsf) | 3404 | 1245 | 1783 | 9.53 | 647 | 1526 | | 8.91 | |  |
| 86 | T3L08(0-3 cmbsf) | 17924 | 2640 | 6393 | 8.56 | 434 | 1179 | | 7.68 | |  |
| 87 | T3L08(6-9 cmbsf) | 5119 | 1211 | 3247 | 8.38 | 471 | 1488 | | 7.80 | |  |
| 88 | T3L08(12-15 cmbsf) | 19355 | 2938 | 6693 | 8.46 | 446 | 1379 | | 7.55 | |  |
| 89 | T3L08(18-21 cmbsf) | 28792 | 4043 | 8575 | 9.08 | 483 | 1304 | | 8.01 | |  |
| 90 | T3L14(0-2 cmbsf) | 11619 | 2249 | 6179 | 8.80 | 480 | 1487 | | 7.96 | |  |
| 91 | T3L14(4-6 cmbsf) | 14257 | 2673 | 6818 | 8.49 | 466 | 1532 | | 7.57 | |  |
| 92 | T3L14(8-10 cmbsf) | 7936 | 1862 | 5048 | 8.62 | 478 | 1471 | | 7.80 | |  |
| 93 | T3L14(12-14 cmbsf) | 27266 | 3385 | 8218 | 8.63 | 429 | 1235 | | 7.71 | |  |
| 94 | T3L14(16-18 cmbsf) | 5333 | 993 | 2614 | 7.55 | 400 | 1111 | | 7.05 | |  |
| 95 | T3L14(18-20 cmbsf) | 15180 | 2478 | 6113 | 8.44 | 425 | 1420 | | 7.60 | |  |

16S rRNA gene amplicons for 95 sediment layers of 14 sediment samples were sequenced. The observed OTU number, Chao1 and Shannon index were determined at a dissimilarity level of 3% and normalized to the smallest library size (i.e. 1143 reads).
