# Supplementary material for: Spatial variations of microbial communities in abyssal and hadal sediments across the Challenger Deep
Source: PeerJ. 2019 May 17;7:e6961. doi: 10.7717/peerj.6961 (PMC6526897; doi:10.7717/peerj.6961)
Supplement: Supplemental Information 7 — The surface layers (0-2 cmbsf) and all the layers were used for PCoA plot in Fig. 3A and Fig. S4, respectively. group1: the sediment sampling site followed by sediment layer. group 2: the water depth with an indicator for sampling site. N before the number for depth represents the northern slope; S denotes the southern slope; T denotes the trench-axis. [file peerj-07-6961-s007.docx]

**Table S3.** Abbreviations of samples names for PCoA plots.

| number | Sample ID | group1 | group2 |
| --- | --- | --- | --- |
| 1 | DD121 | DD121(0-2 cmbsf) | N5500 |
| 2 | DD121 | DD121(4-6 cmbsf) | N5500 |
| 3 | DD121 | DD121(8-10 cmbsf) | N5500 |
| 4 | DD121 | DD121(12-14 cmbsf) | N5500 |
| 5 | DD121 | DD121(16-18 cmbsf) | N5500 |
| 6 | DMC02 | DMC02(0-2 cmbsf) | N5500 |
| 7 | DMC02 | DMC02(4-6 cmbsf) | N5500 |
| 8 | DMC02 | DMC02(8-10 cmbsf) | N5500 |
| 9 | DMC02 | DMC02(12-14 cmbsf) | N5500 |
| 10 | DMC02 | DMC02(16-18 cmbsf) | N5500 |
| 11 | DD120 | DD120(0-2 cmbsf) | N6700 |
| 12 | DD120 | DD120(4-6 cmbsf) | N6700 |
| 13 | DD120 | DD120(12-14 cmbsf) | N6700 |
| 14 | DD119 | DD119(0-2 cmbsf) | N6000 |
| 15 | DD119 | DD119(12-14 cmbsf) | N6000 |
| 16 | DD119 | DD119(16-18 cmbsf) | N6000 |
| 17 | T1B08 | T1B08(0-2 cmbsf) | N7100 |
| 18 | T1B08 | T1B08(4-6 cmbsf) | N7100 |
| 19 | T1B08 | T1B08(8-10 cmbsf) | N7100 |
| 20 | T1B08 | T1B08(12-14 cmbsf) | N7100 |
| 21 | T1B08 | T1B08(16-18 cmbsf) | N7100 |
| 22 | DD114 | DD114(0-2 cmbsf) | S5500 |
| 23 | DD114 | DD114(4-6 cmbsf) | S5500 |
| 24 | DD114 | DD114(12-14 cmbsf) | S5500 |
| 25 | DD114 | DD114(16-18 cmbsf) | S5500 |
| 26 | T1B06 | T1B06(0-2 cmbsf) | S7000 |
| 27 | T1B06 | T1B06(4-6 cmbsf) | S7000 |
| 28 | T1B06 | T1B06(8-10 cmbsf) | S7000 |
| 29 | T1B06 | T1B06(12-14 cmbsf) | S7000 |
| 30 | T1B06 | T1B06(16-18 cmbsf) | S7000 |
| 31 | T1B09 | T1B09(0-2 cmbsf) | S7100 |
| 32 | T1B09 | T1B09(4-6 cmbsf) | S7100 |
| 33 | T1B09 | T1B09(8-10 cmbsf) | S7100 |
| 34 | T1B09 | T1B09(12-14 cmbsf) | S7100 |
| 35 | T1B09 | T1B09(16-18 cmbsf) | S7100 |
| 36 | T1L06 | T1L06(0-2 cmbsf) | S7800 |
| 37 | T1L06 | T1L06(4-6 cmbsf) | S7800 |
| 38 | T1L06 | T1L06(8-10 cmbsf) | S7800 |
| 39 | T1B10 | T1B10(0-2 cmbsf) | T8600 |
| 40 | T1B10 | T1B10(28-30 cmbsf) | T8600 |
| 41 | T1B10 | T1B10(44-46 cmbsf) | T8600 |
| 42 | T1B10 | T1B10(64-66 cmbsf) | T8600 |
| 43 | T1L10 | T1L10(0-3 cmbsf) | T10000 |
| 44 | T1L10 | T1L10(6-9 cmbsf) | T10000 |
| 45 | T1L10 | T1L10(12-15 cmbsf) | T10000 |
| 46 | T1L10 | T1L10(18-21 cmbsf) | T10000 |
| 47 | T3L11 | T3L11(0-3 cmbsf) | T10000 |
| 48 | T3L11 | T3L11(6-9 cmbsf) | T10000 |
| 49 | T3L11 | T3L11(12-15 cmbsf) | T10000 |
| 50 | T3L11 | T3L11(18-21 cmbsf) | T10000 |
| 51 | T3L08 | T3L08(0-3 cmbsf) | T10000 |
| 52 | T3L08 | T3L08(6-9 cmbsf) | T10000 |
| 53 | T3L08 | T3L08(12-15 cmbsf) | T10000 |
| 54 | T3L08 | T3L08(18-21 cmbsf) | T10000 |
| 55 | T3L14 | T3L14(0-2 cmbsf) | T10000 |
| 56 | T3L14 | T3L14(4-6 cmbsf) | T10000 |
| 57 | T3L14 | T3L14(8-10 cmbsf) | T10000 |
| 58 | T3L14 | T3L14(12-14 cmbsf) | T10000 |
| 59 | T3L14 | T3L14(16-18 cmbsf) | T10000 |

The surface layers (0-2 cmbsf) and all the layers were used for PCoA plot in Fig.3A and Fig. S3 respectively.

group1: the sediment sampling site followed by sediment layer.

group 2: the water depth with an indicator for sampling site. N before the number for depth represents the northern slope; S denotes the southern slope; T denotes the trench axis.
