# Supplementary material for: Spatial variations of microbial communities in abyssal and hadal sediments across the Challenger Deep
Source: PeerJ. 2019 May 17;7:e6961. doi: 10.7717/peerj.6961 (PMC6526897; doi:10.7717/peerj.6961)
Supplement: Supplemental Information 8 — cmbsf: centimeter below seafloor. [file peerj-07-6961-s008.docx]

**Table S4.** Ion concentrations (mg/L) of pore waters from the two trench-axis sediment cores.

| sample | layer(cmbsf) | NO_3_^-^ | SO_4_^2-^ |
| --- | --- | --- | --- |
| T1L10 | 0-3 | 44 | 1160 |
|  | 6-9 | 43 | 981 |
|  | 12-15 | 43 | 1223 |
|  | 18-21 | 44 | 1080 |
| T3L11 | 0-3 | 45 | 503 |
|  | 6-9 | 46 | 527 |
|  | 12-15 | 46 | 525 |
|  | 18-21 | 44 | 542 |

cmbsf: centimeter below seafloor.
